# Improving udder health in crossbred cows in New Zealand using Scandinavian Red sires

**DOI:** 10.5455/javar.2026.m1009

**Published:** 2026-03-05

**Authors:** Hamed Amirpour-Najafabadi, Craig McKimmie, Pavithra Ariyarathne, Hossein Alizadeh, Jonathan Hickford

**Affiliations:** 1 Genetics Department, Samen NZ, Morrinsville 3300, New Zealand; 2 AgResearch, Grasslands Research Centre, Palmerston North 4410, New Zealand; 3 Faculty of Agriculture and Life Sciences, Lincoln University, Lincoln 7647, New Zealand

**Keywords:** Dairy cattle, somatic cell count, udder health, antimicrobial resistance, crossbreeding, Scandinavian Red

## Abstract

**Objectives:** To test whether Scandinavian Red (SR) sires improve udder health (somatic cell score; SCS) in New Zealand crossbred dairy cows without compromising milk solids yield.

**Materials and Methods:** Herd-test records (one test day per cow) from six pasture-based New Zealand herds were analyzed (*n* = 5,294). All cows were daughters of Friesian x Jersey dams and were sired by Friesian (F), Jersey (J), Friesian × Jersey (F × J), or SR bulls. SCS was calculated as ln (SCC/1,000). Linear models estimated sire-breed effects on SCS, adjusting for herd, cow age group, days in milk (linear and quadratic), and milk solids (fat + protein, kg/day). Tukey-adjusted comparisons were used.

**Results:** Sire breed significantly affected SCS (*p* = 0.004). SR-sired cows had the lowest adjusted mean SCS and were significantly lower than F- and J-sired cows; F × J-sired cows were intermediate. Milk solids (kg/day) yield did not differ by sire breed (*p* = 0.12). Older cows, early/late lactation, and herds were associated with higher SCS.

**Conclusions:** Under pasture-based New Zealand conditions, using SR sires on Friesian x Jersey dams was associated with improved udder health (lower SCS) without reducing milk solids yield, supporting SR genetics as a practical option to reduce mastitis risk and antimicrobial use.

## 1. Introduction

Mastitis, an inflammation of the mammary gland, is the most prevalent production disease in dairy cows and a major source of economic loss in New Zealand. It reduces milk yield and quality, elevates somatic cell count (SCC), and incurs substantial treatment and culling costs. Industry estimates put the annual cost of mastitis in New Zealand at approximately NZ$180 million [1]. Despite improvements in management practices, mastitis remains common: For example, a nationwide study reported a lactation incidence of around 11% for clinical mastitis in New Zealand herds [2]. The national average bulk milk SCC during the 2022–2023 season was 150,000–170,000 cells/ml [3], underscoring the need for continued improvements in udder health.

Global concerns about antimicrobial resistance have brought renewed focus to mastitis prevention. The New Zealand Veterinary Association (NZVA) has set a goal that by 2030, antibiotics will no longer be routinely needed to maintain animal health and welfare [4]. Rather than simply reducing antibiotic use, this initiative emphasizes disease prevention, improved diagnostics, selective therapy, and breeding for disease resistance. Although New Zealand’s livestock sector currently has one of the lowest rates of antibiotic usage in the OECD [5], mastitis treatment (during lactation and via prophylactic dry-cow therapy) accounts for a large proportion of antibiotics used in the dairy industry [4, 5]. Improving genetic resistance to mastitis is therefore a national priority to support sustainable dairy farming and antimicrobial stewardship.

Scandinavian countries such as Sweden, Denmark, and Finland provide successful examples of mastitis reduction through both management and genetics. These countries have long prioritized udder health. For example, Swedish national herd data indicate annual clinical mastitis incidence rates below 10%, markedly lower than those reported for New Zealand’s predominantly pasture-based herds [6]. Nordic dairy breeding programs explicitly include udder health traits (such as clinical mastitis and SCC) in selection objectives, leading to gradual but steady genetic improvement in mastitis resistance [7]. Crossbreeding studies in Northern Europe have shown that using Nordic Red sires over Holstein cows can improve the health, fertility, and overall robustness of offspring under a range of farming conditions [8]. Accordingly, the introduction of “Red” genetics into New Zealand dairy herds might enhance mastitis resistance while maintaining productivity.

There are limited data to compare the udder health outcomes of cows sired by local versus imported breeds under New Zealand conditions. This study addresses that gap by examining whether sire breed influences SCC and, by extension, mastitis resistance in crossbred New Zealand dairy cows. The Nordic/SR populations exhibit low, but not zero, genetic variation in udder health traits (e.g., in Norwegian Red, heritability is ≈0.03 for clinical mastitis and ≈0.11 for lactation mean SCS), suggesting the possibility of making genetic gain via selection for low CM and/or SCS [9]. Most SR crossbreeding evaluations have been conducted in intensive, indoor systems in Northern Europe, but New Zealand’s pasture-based, seasonal dairy system imposes different energy balance, infection pressure, and management constraints; hence, validating SR udder-health advantages under NZ conditions fills an important evidence gap. This study addresses that gap by examining whether sire breed influences SCS and, by extension, mastitis resistance in crossbred New Zealand dairy cows.

The objective was to quantify breed-related differences in somatic cell score (SCS, a logarithmic transform of SCC) as an indicator of genetic mastitis resistance. We evaluated SCS across cows sired by SR, J, F, or F × J bulls, while controlling cow age, herd, production level, and stage of lactation. We hypothesized that daughters of SR sires, from a population with intensive selection for health traits, would exhibit lower SCS (better udder health) than daughters of local-breed sires. Identifying sire breeds associated with superior udder health could inform breeding strategies to reduce mastitis and support the NZVA’s vision to reduce antibiotic use by 2030.

## 2. Materials and Methods

### 2.1. Ethical approval

The study analyzed routinely collected herd-test records and did not involve experimental procedures or additional animal handling beyond standard farm practice. Accordingly, animal ethics approval was not required. Data were provided by participating farmers with their permission and analyzed in de-identified form.

### 2.2. Study design and data collection

Data were collected in April 2024 from herd-test records of six commercial dairy herds across New Zealand. Each herd had a mix of cows sired by F, J, F × J, or SR bulls. All cows were progeny of F × J cross dams (no purebred dams), which helped standardize maternal breed background. These six herds were not a random sample of the national population; rather, they were purposively selected because they had participated in a breeding program that used SR semen. This was purposive sampling of herds known to use SR sires. Consequently, generalizations should be made with caution, as participating herds herds may differ from the national average in management or health status. Each cow contributed a single test-day record (the most recent available) for analysis. Cows with missing somatic cell data were excluded, leaving 5,294 cow records in the final dataset. No individual cow was represented more than once, so repeated measures modeling was not required. Basic herd descriptors: Herd sizes ranged from 309 to 2,360 cows. Herds 1, 2, and 6 had a higher proportion of older cows (≥ 5 years old), whereas Herd 3 had relatively fewer older cows. All herds were spring-calving, pasture-based operations with twice-daily milking and similar seasonal management. Each herd conducted at least one herd test during the study period (with some herds tested more than once, though only one record (the last) per cow was used as noted).

### 2.3. Traits and abbreviations

SCC was recorded in cells per milliliter and converted to SCS for analysis. SCS was defined as the natural logarithm of SCC/1,000 (i.e., SCS = ln [SCC/1000]) [10]. Milk solids (kg/day) production was measured as the daily yield of fat plus protein (kg per cow per day) at the herd test. Days in milk (DIM) was the number of days since calving at the time of the herd test. For modeling, DIM was included as both a linear and quadratic term to allow a non-linear lactation curve effect. Categorical variables included in the analysis were sire breed (four levels: F, J, F × J, SR), cow age group (2, 3, 4, or ≥ 5 years old), and herd (1 through 6).

### 2.4. Statistical analysis

A linear regression model (ordinarily, least squares) was fitted to evaluate the effects of sire breed and other factors on SCS. Fixed effects in the model were sire breed (categorical), cow age (categorical), herd (categorical), milk solids (continuous, kg/day), and DIM (continuous, with linear and quadratic terms). Dam of cow was not included as a random effect because individual dam identities were not available (each cow had a unique dam, and all dams were crossbreds of similar breed composition), but the inclusion of cow age and dam breed (crossbred for all) in effect coding helped account for some maternal differences. The model equation can be represented as:


1 \[
{\mathrm{SC}}{{\mathrm{S}}_{{\mathrm{ijkl}}}} = {\beta _0} + {\beta _1}\left({{\mathrm{sire\_bree}}{{\mathrm{d}}_{\mathrm{i}}}} \right) + {\beta _2}\left({{\mathrm{ag}}{{\mathrm{e}}_{\mathrm{j}}}} \right) + {\beta _3}\left({{\mathrm{her}}{{\mathrm{d}}_{\mathrm{k}}}} \right) + {\beta _4}\left({{\mathrm{milksolid}}{{\mathrm{s}}_{\mathrm{l}}}} \right) + {\beta _5}\left({{\mathrm{DI}}{{\mathrm{M}}_{\mathrm{m}}}} \right) + {\beta _6}\left({{\mathrm{DIM}}_{\mathrm{m}}^2} \right) + {{\mathrm{e}}_{{\mathrm{ijkl}}}}
\]

where β_0_ is the intercept, β_1_–β_6_ are regression coefficients for the respective predictors, and e_ijkl_ is the residual error term. For categorical predictors (sire breed, age, herd), reference categories were defined as: F for sire breed, 2-year-old for age, and Herd 1 for herd. Thus, model coefficients for other categories represent the difference in SCS from the reference group. Herd was modeled as a fixed effect because there were only six purposively selected herds, and our interest was to adjust explicitly for their management rather than infer to a broader herd population. We also tested a sire-breed × herd interaction to evaluate whether breed effects differed by herd. The interaction was not significant at *α* = 0.05 and was removed for parsimony (breed-specific LSMeans by herd are still shown in Table 2 for transparency). Continuous predictor coefficients represent the estimated change in SCS per unit increase in the predictor (for milk solids, kg/day; for DIM, per day, with a quadratic term included to capture curvature).

We used age (years) instead of parity because age provides a finer adjustment for maturity and cumulative exposure; in these seasonal NZ herds, albeit age and parity are highly correlated.

Model fitting and statistical tests were conducted using R version 4.3.3 [11]. Model diagnostics confirmed approximate normality and homoscedasticity of residuals. Least-squares means (LSMeans) for SCS by sire breed (overall and within each herd) were estimated from the model and compared using Tukey’s honestly significant difference (HSD) test to adjust for multiple comparisons. Significance was judged at *p* < 0.05 unless otherwise stated.

## 3. Results

### 3.1. SCS model outcomes

Table 1 summarizes the linear model estimates for the fixed effects on SCS. For clarity, Table 1 reports estimated marginal means (LSMeans) of SCS by category (and SE), lower SCS values indicate better udder health. Sire breed had a highly significant influence on SCS (overall F-test *p* = 0.004). Relative to F-sired cows (reference group), cows sired by SR bulls had significantly lower SCS (*p* < 0.001). Jersey-sired cows had higher SCS than SR-sired (*p* < 0.001) and tended to be higher than F × J-sired, but the Jersey vs. F × J difference was not significant. F-sired cows exhibited the highest SCS on average. In contrast, SR-sired cows had the lowest SCS (Tables 1, 2), followed by F × J, then Jersey, with Friesian highest. This ordering is consistent across the overall LSMeans and most herds. Older cow age was associated with higher SCS: cows aged 4 years and ≥ 5 years had greater SCS than 2-year-old cows (*p* < 0.001 for both), with the largest increase in the oldest group (+0.68 SCS for 5+ year-olds; Table 1).

**Table 1.** Linear regression model results for effects of sire breed, age, herd, milk solids (kg/day), and days in milk (DIM) on somatic cell score (SCS). Sire breed is coded with F as the reference, age is coded with 2-year-old as the reference, and herd is coded with Herd 1 as the reference. Significant effects (*p* < 0.05) are bold or denoted by asterisks.

Predictor	Category	Estimate	Std. Error	*p*-value
Intercept	(F, 2-yr, Herd 1; 0 milk, 0 DIM)	5.16	0.17	<0.001***
Sire breed	F × J	+4.98	0.17	< 0.001***
	J	+5.13	0.17	< 0.001***
	SR	+4.78	0.16	< 0.001***
Cow age	3-year vs 2-year	+0.08	0.05	0.102
	4-year vs 2-year	+0.33	0.06	< 0.001***
	≥ 5-year vs 2-year	+0.68	0.05	< 0.001***
Herd	Herd 2 vs Herd 1	+0.25	0.05	< 0.001***
	Herd 3 vs Herd 1	+0.01	0.07	0.877
	Herd 4 vs Herd 1	*+0.16*	0.07	0.015*
	Herd 5 vs Herd 1	+0.21	0.07	0.004**
	Herd 6 vs Herd 1	+0.32	0.07	< 0.001***
Milk solids (kg/day)	per kg	–0.633	0.050	< 0.001***
Days in milk (DIM)	Linear term	–0.011	0.0017	< 0.001***
	Quadratic term (DIM^2^)	+3.2 × 10^–5^	5.5×10^–6^	< 0.001***

Significance codes: ***p < 0.001, **p < 0.01, *p <* 0.05.

Three-year-old cows did not differ significantly from 2-year-olds. Herd effects were evident, with Herds 2, 4, 5, and 6 showing higher SCS than Herd 1 (*p* < 0.05), whereas Herd 3’s mean SCS was not significantly different from Herd 1. Milk solids (kg/day) production had a significant negative relationship with SCS (*β* = –0.633 ± 0.050, *p* < 0.001). In other words, higher-producing cows tend to have lower somatic cell counts. Days in milk showed a non-linear association with SCS: the linear DIM term was negative, and the quadratic DIM term was positive (both *p* < 0.001), consistent with SCS being lowest in mid-lactation. Specifically, SCS was higher in early lactation, reached a minimum in mid-lactation, and rose again in late lactation. This pattern is consistent with mastitis dynamics: SCS tends to be higher in early lactation (fresh cows) and again near dry-off (late lactation), with a mid-lactation nadir [12].

Table 1 also provides detailed model coefficients, standard errors, and significance levels for all predictors. For categorical variables, estimates indicate the difference in SCS from the reference category (F for sire breed, 2-year for age, Herd 1 for herd). For continuous variables, the estimate represents the slope of SCS per unit change in the predictor. Table 1 reports no-intercept baseline coefficients on the ln-SCS scale, and breed comparisons are presented as LSMeans with Tukey tests in Table 2.

**Table 2.** Least-squares mean SCS (± SE) by sire breed overall and within each herd. Different superscript letters within a row indicate a significant difference between sire breed groups for that herd (Tukey-adjusted *p* < 0.05). No letters in a row means no significant differences in that herd.

Herd	F × J (± SE)	F (± SE)	J (± SE)	SR (± SE)
All herds (Overall)	3.49^b^ ± 0.04	3.70^a^ ± 0.04	3.60^ab^ ± 0.07	3.30^c^ ± 0.05
Herd 1	3.40^a^ ± 0.07	3.90^b^ ± 0.09	4.20^a^ ± 0.39	3.10^c^ ± 0.07
Herd 2	3.80^a^ ± 0.05	3.90^a^ ± 0.04	3.80^a^ ± 0.07	3.40^b^ ± 0.07
Herd 3	3.40 ± 0.07	3.70 ± 0.15	4.60 ± 1.08	3.10 ± 0.16
Herd 4	3.60^a^ ± 0.09	4.00^a^ ± 0.10	3.80^a^ ± 0.33	2.90^b^ ± 0.09
Herd 5	4.10^a^ ± 0.41	4.00^a^ ± 0.08	4.20^a^ ± 0.14	3.20^b^ ± 0.13
Herd 6	3.90^a^ ± 0.06	4.00^a^ ± 0.04	4.00^a^ ± 0.05	3.30^b^ ± 0.09

Within each row, values sharing the same letter are not significantly different (*p* ≥ 0.05).

### 3.2. Breed group comparisons

Least-squares mean SCS by sire breed (adjusted for other factors) are presented in Table 2. Overall, SR-sired cows had the lowest SCS (3.30 ± 0.05) among the four groups and significantly lower than all other breeds (*p* < 0.05). The F × J-sired cows had intermediate SCS (3.49 ± 0.04) but did not significantly differ from Jersey-sired cows (3.60 ± 0.07). Jersey- and F-sired cows (3.70 ± 0.04) were not significantly different from each other. Thus, while numerical differences were observed between all groups (in the order SR < F × J < J < F), not all differences were statistically significant after accounting for variation. Lower SCS values indicate better udder health, so these results suggest that SR genetics conferred an advantage in mastitis resistance under the conditions of this study.

We also examined SCS differences by sire breed within each herd (Table 2). In five of the six herds, the SR-sired cows had the lowest SCS compared to contemporaries, with many of those differences being significant (*p* < 0.05). For example, in Herds 2, 4, 5, and 6, SR daughters had significantly lower SCS than the daughters of local breeds (letters a, b, c in Table 2 denote significant differences within rows). Herd 3 showed no significant SCS differences among sire groups, likely due to the smaller number of cows and higher variability in that herd. These within-herd patterns reinforce the overall finding that SR sires tended to produce offspring with superior udder health, although herd-to-herd variation in the magnitude of the effect was evident.

### 3.3. Milk production

Milk solids (kg/day) yield did not significantly differ among the sire breed groups (overall ANOVA *p* = 0.12), and Table 3 reveals the average milk solids (kg/day) production by sire breed. F × J-, J-, and SR-sired cows all produced roughly 1.50–1.52 kg of milk solids per day on the test (not significantly different from each other). F-sired cows averaged slightly less (1.45 ± 0.008 kg/day), but the 95% confidence intervals overlapped with those of the other groups, indicating this difference was not statistically meaningful. In practical terms, SR-sired cows performed on par with the high-producing Jersey and crossbred-sired groups, and the modest production gap for F-sired cows was not significant. These results suggest that the genetic gains in udder health associated with SR sires were achieved without any penalty to milk solid production under the study conditions.

**Table 3.** Least-squares means for milk solids production (kg/day) by sire breed. Values in parentheses are the 95% confidence interval for each mean. No significant differences in milk solids (kg/day) yield were detected between sire breed groups (*p* > 0.05) for all pairwise comparisons.

Sire breed	Milk solids (kg/day)	95% CI (Lower–Upper)
F × J	1.52 ± 0.0099	1.50 – 1.53
F	1.45 ± 0.0081	1.44 – 1.47
J	1.51 ± 0.0121	1.48 – 1.53
SR	1.50 ± 0.0174	1.47 – 1.54

## 4. Discussion

Crossbreeding remains a strategic tool for enhancing key traits in dairy cattle by combining the strengths of different breeds. The present study provides evidence that sire breed can affect SCC (and thus mastitis risk) in New Zealand crossbred cows. In particular, daughters of SR bulls had the lowest somatic cell counts among the groups tested, suggesting a genetic contribution from the SR sires toward improved udder health. This finding is consistent with the selection history of the SR and related Nordic Red breeds, which have been intensively selected for low mastitis and SCC as part of their national breeding goals [13, 14]. The SR advantage in our study persisted even though most SR-sired cows were from F × J dams (meaning those cows also benefited from some maternal heterosis). The fact that SR-sired cows outperformed others in udder health despite similar maternal backgrounds indicates a true sire breed effect attributable to favorable alleles in the SR genetics. Prior research has shown that Nordic Red populations harbor genetic variants that confer enhanced mastitis resistance [14].

Our results align with those reports and demonstrate the potential for those benefits to be realized under New Zealand grazing conditions. The SR advantage may partially reflect the genetic architecture of mastitis resistance. Multiple GWAS and syntheses identify stable signals for mastitis/SCS on BTA6 near GC (vitamin-D-binding protein), and both sequence-level variation and a 12-kb enhancer copy-number variant at GC have been linked to resistance in Nordic and other dairy populations. Broader meta-analyses continue to refine candidate loci and pathways involved in immune defense and udder tissue integrity [15]. A modest reduction in SCS is consistently associated with lower clinical mastitis risk and improved farm outcomes, although the exact magnitude varies by herd and methodology. Consequently, lowering SCS via breeding (as observed for SR progeny here) would support the need for fewer antibiotic treatments, less discarded milk, and reduced culling for mastitis [16]. The better udder health of SR-sired cows did not come at the expense of production. The milk solid (kg/day) yields of SR-sired cows were statistically equivalent to those of Jersey- and crossbred-sired cows and only marginally (and non-significantly) higher than those of the F-sired cows. This is an encouraging outcome, as it suggests farmers could improve herd health through crossbreeding with SR bulls without sacrificing milk output. In New Zealand’s seasonal, pasture-based dairying system, traits like fertility and udder health are key drivers of profitability and sustainability. The observed negative phenotypic association between milk solids (kg/day) production and SCS in this study further supports the notion that it may be possible to select or breed for improvements in both productivity and disease resistance simultaneously. Similar concurrent gains in production and health traits have been achieved in Nordic dairy cattle populations through balanced breeding strategies [14]. Our findings provide a local example reinforcing the observation that high milk production and good udder health are not mutually exclusive goals in dairy breeding. Economically, the 0.40-unit lower adjusted mean SCS observed for SR-sired cows compared with Friesian-sired cows (Table 2) could translate into meaningful reductions in mastitis-related costs (treatment, discarded milk, and culling), which are substantial in New Zealand [1].

The pattern of SCS across lactation in this study (lowest in mid-lactation, higher in early and late lactation) was expected and mirrors known mastitis risk patterns. It highlights the importance of tailored herd management during the transition (calving) period and during the approach to dry-off, when cows are most vulnerable to intramammary infections. The significant quadratic DIM effect on SCS underlines this nonlinear risk. It reinforces management recommendations such as careful peripartum monitoring and selective dry cow therapy to prevent mastitis during these critical periods.

Consistent with previous research, older cows in our study had higher SCS on average [17]. That is, cows in their fourth lactation (and beyond) had markedly elevated levels compared to first- and second-lactation animals. This age effect is commonly attributed to the cumulative exposure of older cows to pathogens, as well as to possible immunosenescence or a buildup of chronic infections. The implication for farmers is that mastitis prevention efforts (e.g., teat sealants at dry-off, closer monitoring) are particularly important for older cows in the herd. It also suggests that genetic evaluations for mastitis resistance might benefit from accounting for age to distinguish truly resistant cows from those simply early in their productive life.

We observed significant herd-to-herd variation in SCS levels, even after adjusting for fixed effects. Herd 3 had no significant differences between sire groups, potentially due to its smaller sample size and higher variance. This highlights that management and environmental factors can modulate genetic effects. Herd 3 might have had uniformly low mastitis pressure or excellent overall management, which masked differences. Alternatively, this herd’s data may have been too limited to detect differences. In general, our results should be interpreted in the context that the herds were not a random representation of New Zealand farms, as they were selected for having SR-sired progeny. Nonetheless, they did encompass a range of environments (different regions and management systems), lending credibility to the broader applicability of the findings.

Beyond udder health, SR genetics may confer additional benefits in fertility and overall robustness. Other studies have reported that crossbreeding with Norwegian/SR cattle can improve reproductive performance and reduce the incidence of postpartum disorders in Holstein-based herds [18]. This broader impact is likely due to both heterosis (hybrid vigor) and the favorable genes for fitness traits that have been concentrated in the Nordic breeds.

Incorporating SR sires into a crossbreeding program provides a genetic infusion that can help reduce inbreeding depression. Even though we did not calculate individual inbreeding coefficients in this study, the principle holds that using an unrelated breed will increase heterosis in the offspring. According to New Zealand Animal Evaluation Limited (NZAEL) records, the average crossbred cow in New Zealand has an inbreeding coefficient of around 2.6%, whereas the SR breed has an average inbreeding of roughly 1%. Accordingly, mating local crossbred cows to SR bulls could achieve 100% outcrossing, thereby maximizing heterosis for low-heritability fitness traits such as mastitis resistance and fertility. Crossbreeding with a genetically distinct population, such as SR, not only boosts heterosis but also helps maintain genetic diversity in the national herd, which is beneficial for long-term resilience and adaptability.

In the future, the benefits of using SRs may become even more pronounced in subsequent generations. The first-cross SR daughters in this study were mostly F × J × SR; their offspring (second-generation SR-crosses) could further improve udder health if mated to complementary breeds, as favorable alleles and hybrid vigor may accumulate. Implementing a structured crossbreeding program (e.g., rotating SR, Friesian, and Jersey sires) would maintain heterosis while leveraging SR’s health traits. Indeed, international studies on three-breed rotational crosses (including Viking Red genetics) have reported extended longevity and health in crossbred cows compared to pure Holsteins [19]. Future research in New Zealand should track these SR-cross descendants to confirm sustained improvements in mastitis resistance and overall productivity. It is important to recognize the limitations of this preliminary study. The data came from six herds known to have used SR genetics, which were not randomly selected. This may introduce selection bias; for example, farmers who experiment with SR sires might also be particularly proactive in herd management, which could influence mastitis outcomes. Caution is needed in generalizing the results to all New Zealand herds.

Additionally, because individual dam IDs were not available, we could not separate maternal genetic effects or common environmental dam effects from the sire breed effect. All cows had F × J cross-dams, which standardize breed background to some degree, but subtle differences in dam lines or heterosis could still play a role. Future research using structured experimental matings or larger industry datasets (with pedigree or genomic data) would help validate these findings. Such studies should also examine correlated effects on other economically important traits, including female fertility, longevity, and calving ease, to ensure that improving udder health through SR crossbreeding does not negatively impact those traits. Residual confounding from management cannot be fully excluded even with herd-fixed effects; thus, genetic and management contributions should be interpreted jointly.

## 5. Conclusions

This study demonstrates that crossbreeding New Zealand dairy cows with SR sires can lead to improved udder health, as evidenced by significantly lower somatic cell counts in SR-sired cows compared to those sired by F or J bulls. Importantly, the health advantage of SR genetics was achieved without any reduction in milk solids (kg/day) production, indicating that farmers can gain mastitis resistance benefits without sacrificing yield. These findings support the use of SR (and similar Nordic) breeds as a viable strategy to enhance animal health and welfare in New Zealand’s predominantly crossbred dairy herds. By lowering mastitis incidence, SR crossbreeding can reduce the need for antibiotic treatments, thereby contributing to national goals for prudent antimicrobial use. It also offers a way to increase genetic diversity and combat inbreeding in the dairy population. In an industry increasingly focused on sustainability and animal health, the incorporation of robust genetics from overseas breeds, such as the SR, could play a valuable role. Further long-term studies are recommended to monitor the performance of SR-cross cows over their lifetimes, including reproductive performance and survival, to better quantify the cost-benefit of this crossbreeding approach under New Zealand conditions.

## Data Availability

The data presented in this study are available from the corresponding author upon reasonable request.

## List of abbreviations

AMR, antimicrobial resistance; DIM, days in milk; F, Friesian; F × J, Friesian × Jersey; GC, group-specific component (vitamin D-binding protein gene); GWAS, genome-wide association study; HSD, honestly significant difference; J, Jersey; LSMeans, least-squares means; MPI, Ministry for Primary Industries; NTM, Nordic Total Merit; NZ, New Zealand; NZVA, New Zealand Veterinary Association; OECD, Organization for Economic Co-operation and Development; QTL, quantitative trait locus; SCC, somatic cell count; SCS, somatic cell score; SR, Scandinavian Red.
